# Sensory Quality of Essential Oils and Their Synergistic Effect with Diatomaceous Earth, for the Control of Stored Grain Insects

**DOI:** 10.3390/insects10040114

**Published:** 2019-04-20

**Authors:** Erika Carla Pierattini, Stefano Bedini, Francesca Venturi, Roberta Ascrizzi, Guido Flamini, Rossella Bocchino, Jessica Girardi, Paolo Giannotti, Giuseppe Ferroni, Barbara Conti

**Affiliations:** 1 Department of Agriculture, Food and Environment, University of Pisa, via del Borghetto 80, 56124 Pisa, Italy; erika.pierattini@agr.unipi.it (E.C.P.); francesca.venturi@unipi.it (F.V.); rossellabocchino@gmail.com (R.B.); jessica.girardi91@gmail.com (J.G.); paolo.giannotti@unipi.it (P.G.); giuseppe.ferroni@unipi.it (G.F.); barbara.conti@unipi.it (B.C.); 2 Department of Pharmacy, University of Pisa, Via Bonanno 6, 56126 Pisa, Italy; roberta.ascrizzi@gmail.com (R.A.); guido.flamini@farm.unipi.it (G.F.)

**Keywords:** essential oil, inert dusts, sensory quality, stored grain insect pest, diatomaceous earths

## Abstract

Essential oils (EOs) have gained increasing interest as a low-toxic, eco-friendly alternative to synthetic repellents and insecticides against insect pests. However, they have scarce practical application in the protection of stored grain because of their limited efficacy and their interference with the organoleptic properties of the grain. In this study, we evaluated the olfactory profile of the EOs of *Foeniculum vulgare*, *Pistacia lentiscus*, and *Ocimum basilicum,* and their toxicity against the main stored grain pest *Sitophilus granarius*. Trained assessors identified *O. basilicum* and *F. vulgare*, as more suitable than the *P. lentiscus* EO for the wheat treatment. In laboratory tests, the most toxic EO was the *P. lentiscus* (LC_50_ = 36.36 μL∙kg^−1^) while, the least toxic, was the *F. vulgare* one (LC_50_ = 77.59 μL∙kg^−1^). The EOs were also tested combined with diatomaceous earths (DEs) showing synergistic effects (co-toxicity coefficient values ranging from 1.36 to 3.35 for *O. basilicum* and *F. vulgare* EOs, respectively). Overall, *O. basilicum* resulted as the best EO for the wheat treatment, considering its insect toxicity and olfactory profile. In real storage conditions, the wheat co-treated with *O. basilicum* EO and DEs showed a significantly lower mean infestation (1.5 insect kg^−1^) than the non-treated wheat (7.0 insect kg^−1^).

## 1. Introduction

Insects represent a serious threat for the stored food. In particular, in cereals and pulses, insect pests cause direct damage by feeding on the seeds, and indirect damage by causing “pockets” of heat and moisture, in the mass of the stored grain. By consequence, they also promote the development of mycotoxigenic fungi, premature sprouting, and the compaction into solidified masses of unusable grain [1]. To date, the control of foodstuff pests is based on synthetic insecticides and fumigants and poses questions about the food and environmental safety, in addition to the insurgence of insecticide resistance among pest populations [2]. Alternative treatments, such as modified atmospheres, low temperatures, microbial insecticides, plant extracts, and inert dusts are currently of interest for the control of stored product pests, in compliance with the principles of Integrated Pest Management [3,4,5,6].

Because their low toxicity to mammals [7] and high biodegradability, essential oils (EOs) of aromatic plants are regarded as very promising substances for the formulation of low-toxic, eco-friendly pest control products [8,9,10,11,12,13]. However, despite their efficacy and low toxicity, EOs application in practice is still limited because of their high volatility, composition variability, and strong smell. In fact, EOs olfactory traits may interfere with the sensory characteristics of the treated foodstuff, resulting in products that are not appealing to the consumers [14].

Inert dusts such as diatomaceous earths (DEs), composed of amorphous SiO_2_, derived from fossilized diatoms, are already registered and commercialized as grain protectant. However, to be effective they need to be applied at high dosages (0.5–3.5 g∙kg^−1^ grain), causing increase of the grain bulk weight, decrease in quality, and interferences with machinery functioning [15].

Interestingly, it has been observed that DEs efficacy against insect pests can be enhanced by co-administration with plant extracts and EOs [16,17,18,19], thus allowing the reduction of DEs doses to be applied to the stored products [19,20]. Therefore, in this study we firstly evaluated the sensory quality of the EOs extracted from *Foeniculum vulgare* Mill. (Apiaceae), *Ocimum basilicum* L. (Lamiaceae), and *Pistacia lentiscus* L. (Anacardiaceae). Then, we determined the toxicity of the three EOs alone and in combination with DEs against the weevil *Sitophilus granarius* (L.) (Coleoptera Curculionidae), in laboratory conditions and, finally, the EO that showed the best combination of smell profile and toxicity against *S. granarius* was evaluated alone and in combination with DEs in a medium-size experiment for the control of grain insect pests under real storage conditions.

## 2. Materials and Methods

### 2.1. Inert Dust

The inert dust used in the research was the DEs “Silicosec” supplied by Biogard (CBC Group, Grassobbio, BG, Italy), containing 50% of particles smaller than 9.46 μm and registered, in Italy, as crop protectant.

### 2.2. Essential Oils and Chemical Analyses

The EO of *F. vulgare* was purchased from KOS srl (Carmignano, PO, Italy); the EO of *O. basilicum*, methyl chavicol type, was purchased from Sigma-Aldrich (Milan, MI, Italy); the EO of *P. lentiscus* was purchased from Efit srl (Terni, TR, Italy). The EOs were diluted to 0.5% in HPLC-grade *n-*hexane and then injected into a gas chromatography–mass spectrometry (GC–MS) apparatus. Gas chromatography–electron impact mass spectrometry (GC–EIMS) analyses were performed with an Agilent 7890B gas chromatograph (Agilent Technologies Inc., Santa Clara, CA, USA) equipped with an Agilent HP-5MS (Agilent Technologies Inc.) capillary column (30 m × 0.25 mm; coating thickness 0.25 μm) and an Agilent 5977B single quadrupole mass detector (Agilent Technologies Inc.). Analytical conditions were as follows: Injector and transfer line temperatures 220 and 240 °C, respectively; oven temperature programmed from 60 to 240 °C at 3 °C/min; carrier gas helium at 1 mL/min; injection of 1 μL (0.5% HPLC grade *n*-hexane solution); split ratio 1:25. The acquisition parameters were as follows: Full scan; scan range: 30–300 *m/z*; scan time: 1.0 s. Identification of the constituents was based on a comparison of the retention times with those of the authentic samples, comparing their linear retention indices relative to the series of *n*-hydrocarbons. Computer matching was also used against commercial (NIST 14 and ADAMS) and laboratory-developed mass spectra library built up from pure substances and components of known oils and MS literature data [21,22,23,24,25,26].

### 2.3. Essential Oils Sensory Characterization

The smell profiles of the EOs of *F. vulgare*, *O. basilicum*, and *P. lentiscus,* alone and in combination with wheat grains (50 μL∙100 g^−1^), were evaluated by a trained panel composed by 12 assessors (“expert panel” of the Department of Agriculture, Food and Environment (DAFE) of University of Pisa) [27,28]. All assessors had previous experience in sensory descriptive analysis. The assessors were provided with a specifically developed sensorial sheet consisting of a non-structured, parametric, descriptive scoring chart. The panelists described the main odors characterizing each sample on the basis of defined descriptors in terms of smell “Intensity”, “Persistency”, and “Pleasantness”, as hedonic parameters, and they gave a score for each of them, where the minimum level was 0 and the maximum was 10. The blind smell test was performed in the morning, in a well-ventilated quiet room and in a relaxed atmosphere. Each panelist was provided with a 2 × 2 cm filter paper soaked with 50 μL of an unknown EO and with a clean 100 mL glass beaker containing 100 g of wheat grain treated with an unknown EO. To avoid cross contamination, the six samples were assessed separately in the same morning (15 min waiting between two assessing).

### 2.4. Laboratory Toxicity Bioassays

Ten unsexed adults of *S. granarius* (1–3 days old) were introduced into glass jars (300 ml) filled with 150 g of either treated or non-treated (control) maize (15% r.h.). The EO-treated wheat was prepared by mixing the wheat with ethanolic solutions of the EOs in order to obtain a final concentration of 0, 25, 50, and 100 μL∙kg^−1^ grain. The EO plus DEs-treated wheat was prepared by mixing the wheat with a formulation obtained by direct absorption of the EOs onto DEs in 1:1 (w:w) proportion. The final concentrations of the EO plus DEs-treated wheat were 0, 20, 40, and 60 mg∙kg^−1^ grain. The experiments were replicated four times. The sealed jars were kept at room temperature (25 ± 1 °C), 65% relative humidity, in the dark. The number of dead insects in each jar was checked after nine days. 

### 2.5. Medium-Scale Toxicity Bioassays

A medium-scale toxicity bioassay was set up in at the Cooperativa Maidicola (Sovicille, SI, Italy). Twelve mini-silos containing 250 kg of insect-free wheat grain each were placed in the grain storage plant of the cooperative at the base of the silos. Fifty adults of *Sitophilus granarius* (Coleoptera Curculionidae), *Rhyzopertha dominica* (Coleopera Bostrichidae), *Cryptolestes ferrugineus* (Coleoptera Cucujidae), *Tribolium castaneum* (Coleoptera Tenebrionidae), and *Oryzaephilus* spp. (Coleoptera Silvanidae) adults were put in each mini-silo to assure a basal level of infestation. The insects were reared under laboratory conditions (24 °C, 45–65% r.h., and in the dark) at the Department of Agriculture, Food and Environment of the University of Pisa. Plastic boxes (20 × 27 × 11 cm) containing broken corn and wheat, covered by a nylon net allowing air exchange, were used for the rearing. The wheat in the mini-silos was treated with: *O. basilicum* EO, DEs, and DEs plus *O. basilicum* EO (1:1, w:w) formulation to obtain a final concentration of 130 μL∙kg^−1^ grain for the EO of *O. basilicum* and of 130 mg∙ kg^−1^ grain for the DEs and DEs plus *O. basilicum* EO formulation. To assure a uniform distribution, after the addition of the substances, the grain of each mini-silo was mixed by a mortar mixer (Paint/Mortar Mixer mod. TC-MX 1400 E, Einhell Italia srl, Binago, CO, Italy). Three replicates for each treatment and non-treated wheat as control were performed. The trial was performed from August 2017 to March 2018. The insect infestation of the wheat was checked after 30, 60, 90, 120, 150, and 240 days, taking 5 wheat samples (in the center and in each of the 4 corners, 10 cm from the edge) from each mini silo (about 500 g of wheat). The samples were collected using a mechanical sampler for cereals (PNS sampler, IROM Italia srl, Brugherio, MB, Italy). The samples were transferred in laboratory and the number of alive insects within each sample was assessed by sieving. Only *R. dominica*, *C. ferrugineus*, *T. castaneum*, *Oryzaephilus* spp., and *Sitophilus* spp. adults were counted.

### 2.6. Statistics and Data Analyses

Data of the assessments of the smell profiles of EOs were processed by one-way ANOVA with the EO treatment as factor. When needed, data were normalized by arcsine transformation. Averages were separated by Tukey’s multiple comparison test (*p* < 0.05). For the toxicity bioassay, median lethal concentration (LC_50_) was calculated by Log-probit regression. LC_50_ values were then pairwise compared by relative median potency (RMP) analysis. The synergistic effects between EOs and DEs was evaluated by co-toxicity coefficient (CTC) according to Sun and Johnson [29]: CTC = LC_50_ of DEs /LC_50_ of formulation (EO + DEs). If the result of calculation was higher than 1, the effect was considered as synergistic, whereas if less than 1, the effect was considered as antagonistic. 

Data of the medium-scale toxicity bioassays were analyzed by a mixed model two-way analysis of variance with repeated measures (RM-ANOVA) followed by Tukey’s multiple comparisons. Wheat treatment and time were considered as fixed effects with their interaction. Greenhouse-Geisser correction was applied in case of violation of Mauchly’s test of sphericity (*p* < 0.05). Non-normally distributed data were square-root transformed before statistical analysis. Data were considered statistically different when *p* < 0.05. Analyses were performed with the SPSS 22.0 software (SPSS Inc., Chicago, IL, USA).

## 3. Results

### 3.1. Essential Oils Composition

The composition of the EOs are reported in Table 1 A total of 49 compounds were identified among the three samples (Supplementary Materials Table S1).

The *P. lentiscus* EO composition was dominated by monoterpene hydrocarbons, which accounted for more than 90% of the totality of identified compounds: α-pinene, in particular, was the most abundant compound in the EO, representing over 65% of the total. Among this chemical class, myrcene and β-pinene followed, showing relative abundances of 16.4% and 4.9%, respectively. Regarding the other compounds, only limonene, β-caryophyllene, camphene, o-methyl anisole, and trans-verbenol showed a relative content over 1.0%.

The *O. basilicum* EO exhibited a methyl chavicol chemotype (78.5%), with a predominance of phenylpropanoids (over 80%) in its total composition. Among the other compounds, trans-α-bergamotene and 1,8-cineole exhibited a relative presence over 4%, accounting for 4.6% and 4.3%, respectively.

The *F. vulgare* EO presented (E)-anethole as the predominant (>80%) compound: as in *O. basilicum*, phenylpropanoids represented the most abundant chemical class of compounds of this EO composition. Among terpenes, limonene showed a relevant relative abundance, accounting for 9.3% of the total, followed by fenchone (2.5%).

### 3.2. Essential Oils Sensory Characterization

**Figure 1:**
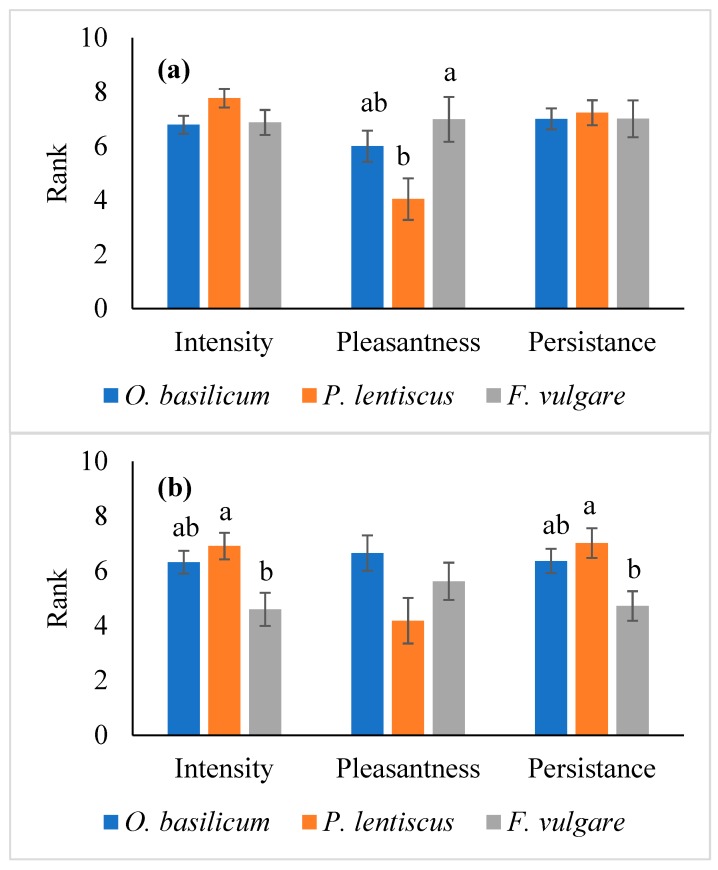
Essential oils (EOs) and EOs-aromatized wheat sensorial description. Sensorial descriptors were ranked by panel components on a 0 to 10 scale. Histograms represent the mean of the ranks. (**a**) sensorial descriptors of the EO of *Foeniculum vulgare*, *Ocimum basilicum*, and *Pistacia lentiscus*; (**b**) sensorial descriptors of the EOs-aromatized wheat. Data represent the mean values of the smell ranks as assessed by the panelists. Bars represent the standard error. Different letters indicate significant difference among the treatments according to Tukey’s b post-hoc test (*p* < 0.05).

All the tested EOs were characterized by the same “Intensity” and “Persistency” (F_2,28_ = 2.030, *p* = 0.150 and F_2,28_ = 0.063, *p* = 0.939, respectively), while the “Pleasantness” resulted as significantly different (F_2,28_ = 3.958, *p* < 0.031) with *F. vulgare* Eos that showed the highest rank values and *P. lentiscus* EO showing the lowest (Figure 1a). When added to wheat, the EOs showed a significant difference for the smell “Intensity” and “Persistency” (F_2,28_ = 5.041, *p* = 0.013 and F_2,28_ = 4.790, *p* = 0.016, respectively). The EO of *F. vulgare* resulted as less intense and persistent in comparison with the EO of *P. lentiscus* (Figure 1b), while no significant differences were recorded for the smell “Pleasantness” (F_2,28_ = 2.913, *p* < 0.071). In general, among the tested EOs, *O. basilicum* and *F. vulgare* EOs showed the best olfactory traits, both alone and in combination with wheat. Conversely, the smell profile of *P. lentiscus* EO was characterized by the presence of “Chemical” off-flavors, as indicated by panelists (Table 2). 

### 3.3. Laboratory Toxicity Bioassays

All the tested EOs showed a clear insecticidal activity against *S. granarius* adults. According to the median lethal concentration (LC_50_) of EOs, calculated by probit regression, *P. lentiscus* exhibited the highest efficacy (LC_50_ = 36.36 μL∙kg^−1^ grain) against *S. granarius* among the three tested EOs, while the EO of *F. vulgare* had the lowest toxicity (LC_50_ = 77.59 μL∙kg^−1^ grain). The comparison of the toxicity values by RMP analysis indicated that the differences of effectiveness among the three EOs were statistically significant. (*O. basilicum vs. P. lentiscus* RMP = 3.58 (2.32–6.09); *O. basilicum vs. F. vulgare* RMP = 0.57 (0.40–0.77); *P. lentiscus vs. F. vulgare* RMP = 0.16 (0.08–0.27)). When the EOs were assayed in combination with DEs, the three EOs efficacy increased substantially, showing synergistic effects (CTC > 1). In particular, the strongest synergistic effect was observed in the *F. vulgare* plus DEs treatment (CTC = 3.35). When administered in combination, the highest toxicity was observed for the *P. lentiscus* plus DEs formulation (LC_50_ = 24.79 mg∙kg^−1^ grain), while the lowest was for the *F. vulgare* plus DEs formulation (LC_50_ = 23.18 mg∙kg^−1^ grain) (Table 3). However, according to RMP analyses, such differences were not statistically significant [*O. basilicum* + DEs *vs. P. lentiscus* + DEs RMP = 1.22 (0.89–1.69); *O. basilicum* + DEs *vs. F. vulgare* + DEs RMP = 1.15 (0.83–1.64); *P. lentiscus* + DEs *vs. F. vulgare* + DEs RMP = 0.95 (0.66–1.35)].

### 3.4. Medium-Scale Toxicity Bioassays

Since according to the sensory characterization and the laboratory toxicity bioassays the *O. basilicum* EO was characterized by high ranks of hedonic parameters (Figure 1), and a higher toxicity than the EO of *F. vulgare* (Table 3), it was chosen for the medium-size experiments.

**Figure 2:**
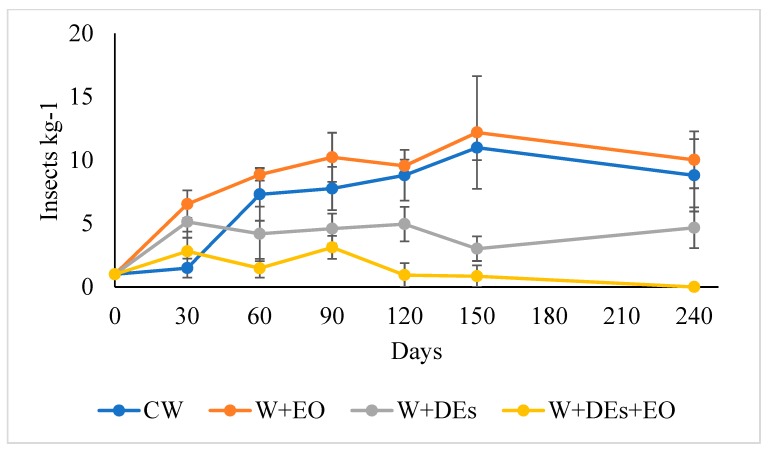
Temporal evolution of the beetles infestation of the stored in the mini-silos during the trial (240 day). CW, control wheat; W + EO, wheat treated with the essential oil of *Ocimum basilicum*; W + DEs, wheat treated with diatomaceous earths; W + Des + OE, wheat treated with *O. basilicum* essential oil and diatomaceous earths. Data represent the mean number of adults kg^−1^ of grain. Bars represent the standard error.

**Figure 3:**
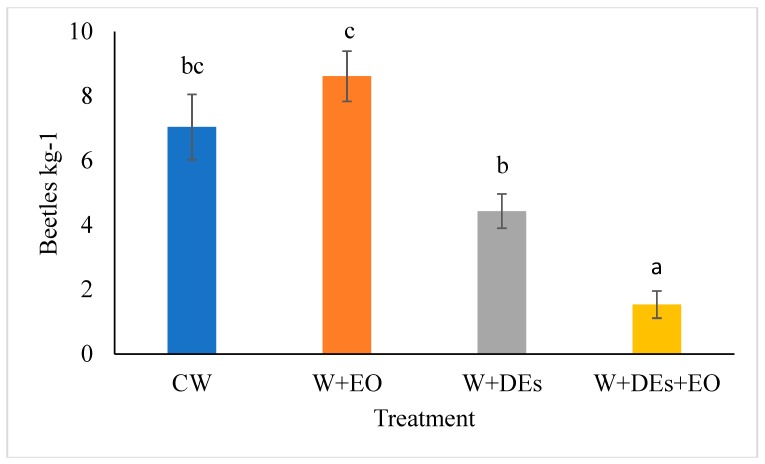
Average beetle infestation of the wheat stored in the mini-silos. CW, control wheat; W+EO, wheat treated with the essential oil of *Ocimum basilicum*; W + DEs, wheat treated with diatomaceous earths; W + DEs + OE, wheat treated with *O. basilicum* essential oil and diatomaceous earths. Data represent the mean number of adults kg^−1^ of grain. Bars represent the standard error. Different letters indicate significant differences among the treatments according to Tukey’s b post-hoc test (*p* < 0.05).

The effectiveness of the DEs and of the EO of *O. basilicum*, alone and in combination, were also evaluated in a medium-scale assay. An estimation of the number of alive adult beetles per kilogram of wheat is shown in Figure 2. RM-ANOVA showed that the grain infestation differed significantly among the treatment (F_3,8_ = 10.531, *p* = 0.004). On average, the number of insects recorded in the wheat of the mini-silos during the period of observation (240 day) varied from 8.61 ± 0.78 to 1.53 ± 0.42 insects kg^−1^ for *O. basilicum* EO and DEs + EO, respectively (Figure 3), with differences among treatments statistically significant (F_3,8_ = 10.531, *p* = 0.004, mixed-model RM-ANOVA). DE combined with EO treatments lead to a three times lower grain infestation than the DEs only treatment (Figure 3). 

## 4. Discussion

Despite their high potential for insect repellency and food protection [8,30,31], treatment of grain with EOs may be limited due to undesirable changes in organoleptic quality of food [32,33]. In this trial, the EOs sensory characterization by the trained panel of assessors indicated a different smell profile for the three tested EOs and of the EOs-aromatized wheat. In general, *P. lentiscus* EO was characterized by a less appealing smelling profile in comparison with *O. basilicum* and *F. vulgare* EOs, whose properties are widely exploited in aromatherapy [34].

The description of the main sensory attributes associated to the three EOs was consistent with their chemical composition as detected by GC-MS. In particular, the odor expression of the EO of *O. basilicum* can be well explained by the presence of methyl chavicol (sweet, phenolic, anise, harsh, spice, green, herbal, minty) and 1,8-cineole (eucalyptus, herbal, camphor, medicinal, with cooling effect) [35]. Besides, the smell expression of *P. lentiscus* is well represented by the odor character generally attributed to α-pinene (woody, piney and turpentine-like, with a slight cooling camphoraceous nuance and a fresh herbal lift), β-pinene (cooling, woody, piney and turpentine-like with a fresh minty, eucalyptus, and camphoraceous note with a spicy peppery and nutmeg nuance), Myrcene (terpy, herbaceous, woody with a rosy celery and carrot nuances), and β-caryophyllene (sweet, woody, spice, clove, dry nuances). Furthermore, the off-flavor specifically attributed to *P. lentiscus* can be due to the presence of o-methyl anisole (naphthyl, camphoraceous, phenolic, and woody with a salicylate nuance), α-campholenal (herbal, green, woody nuances) together with camphene (camphoraceous, cooling, piney woods with terpy nuances). Finally, (E)-anethole, together with anisic ketone and limonene can be assumed as the chemical compounds that characterize, specifically, the odor of *F. vulgare*, as they were not detected in the volatile fraction of the other two EOs tested. Their presence perfectly justifies the description of the smell provided by judges during panel test of *F. vulgare*, as their odor expression can be described by referring to: sweet, anise, licorice, and mimosa for (E)-anethole; citrus, orange, fresh, sweet for limonene; and sweet, fruity spicy, with anise balsamic nuances for anisic ketone.

Beside the differences in the sensory quality, we also found differences in the insecticidal activity of the three EOs alone and when administered in combination to DEs. In general, we observed a good insecticidal activity of the tested EOs with *P. lentiscus* EO, which exhibited the highest toxicity (LC_50_ = 36.36 μL∙kg^−1^ grain), and the EO of *F. vulgare* exhibited the lowest one (LC_50_ = 77.59 μL∙kg^−1^ grain). Such bioactivity is in line with a previous study on the bioactivity of the EOs of *P. lentiscus* and *O. basilicum* against foodstuff insect pests. Bachrouch et al. [36] found LC_50_ values of 8.44 and 28.03 μL∙L^−1^ of *P. lentiscus* EO against *Lasioderma serricorne* (Coleoptera Anobiidae) and *T. castaneum*, respectively. Similarly, *O. basilicum* EO was found to be toxic against *Callosobruchus maculatus* (Coleoptera Bruchidae) (LC_50_ = 75 μL∙kg^−1^) [37], *Oryzaephilus surinamensis* (Coleoptera Silvanidae), and *R. dominica* (LC_50_ = 6.7 and 10 μL∙L^−1^ air, respectively) [38]. On the contrary, very little information is available for the insecticidal activity of *F. vulgare* against foodstuff insect pests. However, similarly to our results, Zhao et al. [39] found a clear toxicity by fumigation of *F. vulgare* EO against the booklouse *Liposcelis bostrychophila* (Psocoptera Liposcelididae) (LC_50_ = 34.1 μL∙L^−1^ air) and Seada et al. [40] and Bedini et al. [9] observed a clear repellent activity of *F. vulgare* EO against *R. dominica*, *S. zeamais*, and *T. confusum,* whose intensity was dependent on the plant chemotype. 

In our experiments we observed a synergistic effect on the toxicity of DEs and all the tested EOs when administrated together. In particular, such synergistic effect was very marked for *F. vulgare* EO, that showed the lowest efficacy among the three EOs tested when administered alone, but the highest efficacy when applied together with Des, with an increase of the toxicity of about three times. A similar synergistic effect between EOs and other bioactive substances has been previously observed for EOs and pyrethroids, as described for the EO of *Elettaria cardamomum* and the pyrethroid lambda-cyhalothrin [41], and for various EOs in combination with inert dusts, such as DEs or kaolin [16,17,19].

In this work, because of its high insecticidal activity coupled with its good sensory quality, the EO of *O. basilicum* was found to be the most suitable for treatment of the grain as protectant against insect pest. However, when tested in the mini silos, the EO of *O. basilicum* alone resulted as the treatment with the highest mean presence of insects, while the grain co-treated with DEs and *O. basilicum* EO showed the lowest mean insect presence during the experiment. Contrary to what was observed in the laboratory, the inefficacy of the EO of *O. basilicum* in the grain storage plant could be due to the dilution of EO vapors in the air, to doses that might have been attractive to the insects. Plant volatiles play an essential role in communication between host plants and herbivorous insects [42,43]. The differential volatilization of the different compound of the EO may have resulted in an attractive effect on the local insect population. A similar insect attraction to *O. basilicum* EO was observed by Górski [44] in greenhouses experiment for the trapping of *Trialeurodes vaporariorum* (Homoptera Aleyrodidae). On the contrary, the enhanced toxicity of the same EO when combined with the DEs could be due to a gradual release, and; therefore, higher persistence of EO volatiles when adsorbed onto the surface of the DEs particles. In line with our observations, previous studies indicated that a co-treatment with EO and DEs have a more pronounced persistence of the insecticidal activity of the two substances administrated alone [20]. The observed enhancement of the effectivity and persistence of the insecticidal activity of the EOs when administrated together with the DEs may be due to the small particles that constitute the Des, whose large surface area may improve the EOs retention. In fact, Ziaee and coworkers [16] observed that such synergistic effect occurs mainly when DEs have particle size smaller than 37 μm. In addition, according to Athanassiou et al. [45] an increased mobility of the insects increases the possibility of picking up dust particles. Similarly, the EO may have enhanced the locomotion of the insects [16] in the mass of the treated grain, thus the abrasion of their protective wax layer by the DEs [46,47] with consequent lethal insect dehydration [48].

## 5. Conclusions

Overall, our results indicate a potential utilization of EOs and DEs for the protection of the stored grain in integrated pest management programs and in the organic agriculture chain. Although the insecticidal properties of EOs and DEs are already known, this research showed that by way of a co-treatment it is possible to enhance the efficacy of the two substances, overcoming some of the problems related to the use of substances individually. In particular, the reduced quantity of the EOs and DEs may reduce the DEs side effects for the workers and machinery and the EOs impact on the olfactory characteristics of the grain. To that regard, future trials will be designed to test how the EO plus DEs treatments may affect the final consumers preferences for bread, pasta, and other products prepared with the treated wheat.

## Figures and Tables

**Table 1:** Chemical composition of the essential oils of *Pistacia lentiscus*, *Ocimum basilicum*, and *Foeniculum vulgare*.

Chemical Classes	Relative Abundance (%)		
	*Pl*	*Ob*	*Fv*
Monoterpene hydrocarbons	91.2	2.8	13.6
Oxygenated monoterpenes	3.8	8.7	2.5
Sesquiterpene hydrocarbons	1.5	6.4	0.1
Oxygenated sesquiterpenes	0.4	1.6	-
Diterpene hydrocarbons	0.2	-	-
Phenylpropanoids	0.3	80.1	81.6
Non-terpene derivatives	1.0	-	2.3
Total identified (%)	98.3	99.7	100.0

l.r.i., linear retention index; EO, essential oil, Pl, P. lentiscus EO, Ob, O. basilicum EO; Fv, F. vulgare EO; -, not detected.

**Table 2:** The main odors that characterized the smell of the essential oils of *Pistacia lentiscus*, *Ocimum basilicum*, and *Foeniculum vulgare*, alone and added to the wheat.

	*O. basilicum*		*P. lentiscus*		*F. vulgare*	
	EO	EO + wheat	EO	EO + wheat	EO	EO + wheat
**Vegetative odors**	mint	mint	mint	mint	anis	anis
	thyme	thyme	thyme	thyme	floral	floral
	caper	caper	caper	caper	fennel	fennel
	licorice	licorice	calamint	calamint	licorice	licorice
	calamint	calamint				
**Spicy odors**	balsamic	balsamic	balsamic	balsamic	sweet	sweet
	spices	spices	spices	resin	balsamic	balsamic
	resin	resin	resin		spices	spices
**Off-flavors**			chemical			

**Table 3:** Toxicity of the essential oils of *Pistacia lentiscus*, *Ocimum basilicum*, and *Foeniculum vulgare*, inert dust and of their combination against adults of *Sitophilus granarius*.

Treatment	CTC ^a^	LC_50_^b^	Slope	Intercept	χ2 (df)	Sig. ^c^
Des ^d^		36.47 (24.84–46.72) ^a^	1.783 ± 0.404	−2.785 ± 0.687	1.912 (1)	0.167
Ob ^e^		43.42 (35.08–52.32)	2.447 ± 0.422	−4.007 ± 0.718	0.036 (1)	0.849
ObDEs ^f^	1.36	26.78 (19.23–32.82)	2.643 ± 0.478	−3.774 ± 0.782	0.007 (1)	0.934
Pl ^g^		36.36 (26.42–51.76)	0.945 ± 0.197	−1.475 ± 0.307	1.089 (3)	0.780
PlDEs ^h^	1.47	24.79 (19.57–29.11)	3.843 ± 0.707	−5.358 ± 1.072	0.627 (1)	0.429
Fv ^i^		77.59 (58.92–120.12)	2.104 ± 0.707	−3.976 ± 1.309	0.033 (1)	0.860
FvDEs ^l^	3.35	23.18 (13.14–29.88)	2.669 ± 0.741	−3.644 ± 1.141	0.003 (1)	0.960

^a^ Co-toxicity coefficient; ^b^ Concentration of the extract that kills 50% of the exposed insects. ^c^ significance of Pearson goodness-of-fit test. ^d^ Diatomaceous earths; ^e^ *Ocimum basilicum* essential oil (EO); ^f^ *Pistacia lentiscus* EO; ^g^ *Foeniculum vulgare* EO; ^f^ *O. basilicum* EO plus DEs; ^g^ *P. lentiscus* EO; ^h^ *P. lentiscus* EO plus DEs; ^i^ *F. vulgare* EO; ^l^ *F. vulgare* EO plus DEs; data are expressed as μL∙kg^−1^ grain for the EOs and as mg∙kg^−1^ grain for the DEs and EO plus DEs treatments.

## Supplementary Materials

The following are available online at https://www.mdpi.com/2075-4450/10/4/114/s1, Table S1: Chemical composition of the essential oils of *Pistacia lentiscus*, *Ocimum basilicum*, and *Foeniculum vulgare*.
