# Supplementary material for: Sensory Quality of Essential Oils and Their Synergistic Effect with Diatomaceous Earth, for the Control of Stored Grain Insects
Source: Insects. 2019 Apr 20;10(4):114. doi: 10.3390/insects10040114 (PMC6523765; doi:10.3390/insects10040114)
Supplement: Supplementary file 1 [file insects-10-00114-s001.pdf]

Table S1. Chemical composition of the essential oils of *Pistacia lentiscus*, *Ocimum basilicum*, and *Foeniculum vulgare*.

Compounds	l.r.i.	Relative abundance (%)		
		Pl	***Ob***	***Fv***
α-pinene	941	66.0	0.3	0.7
camphene	954	1.2	-	-
thuja-2,4(10)-diene	959	0.2	-	-
sabinene	976	0.4	0.2	-
β-pinene	982	4.9	0.5	0.3
myrcene	993	16.4	0.3	0.6
α-phellandrene	1005	-	-	1.9
o-methyl anisole	1007	1.0	-	-
δ-3-carene	1011	-	-	0.1
p-cymene	1027	0.2	-	0.6
limonene	1032	1.8	0.2	9.3
1,8-cineole	1034	-	4.3	-
(*Z*)-β-ocimene	1042	-	-	0.2
(*E*)-β-ocimene	1052	-	1.4	-
fenchone	1087	-	0.2	2.5
perillene	1100	0.3	-	-
linalool	1101	0.9	1.3	-
fenchol	1113	-	0.2	-
α-campholenal	1125	0.6	-	-
trans-pinocarveol	1139	0.4	-	-
camphor	1143	-	0.7	-
trans-verbenol	1144	1.0	-	-
menthone	1154	-	0.1	-
borneol	1165	-	0.2	-
menthol	1173	-	0.5	-
α-terpineol	1189	0.1	0.5	-
myrtenal	1194	0.3	-	-
methyl chavicol	1197	-	78.5	0.9
verbenone	1205	0.2	-	-
endo-fenchyl acetate	1223	-	0.4	-
p-anisaldehyde	1256	-	-	1.2
(*E*)-anethole	1283	0.3	-	80.7
bornyl acetate	1287	-	0.3	-
α-ylangene	1372	0.1	-	-
anisic ketone	1384	-	-	1.1
β-elemene	1392	-	0.4	-
methyl eugenol	1403	-	0.9	-
β-caryophyllene	1420	1.3	0.3	0.1
trans-α-bergamotene	1438	-	4.6	-
α-humulene	1456	0.1	-	-
α-bulnesene	1505	-	0.1	-
trans-γ-cadinene	1513	-	1.0	-
δ-cadinene	1524	-	0.2	-
4-methoxycinnamaldehyde	1570	-	0.7	-
spathulenol	1576	-	0.1	-
caryophyllene oxide	1581	0.4	-	-
1,10-*di*-*epi*-cubenol	1614	-	0.2	-
epi-α-cadinol	1640	-	1.4	-
m-camphorene	1960	0.2	-	-

l.r.i., linear retention index; Pl, Pistacia lentiscus EO, Ob, Ocimum basilicum essential oil (EO), Fv, F. vulgare EO; -, not detected.
